# Data Augmentation-Enhanced Myocardial Infarction Classification and Localization Using a ResNet-Transformer Cascaded Network

**DOI:** 10.3390/biology14101425

**Published:** 2025-10-16

**Authors:** Yunfan Chen, Qi Gao, Jinxing Ye, Yuting Li, Xiangkui Wan

**Affiliations:** 1 Hubei Key Laboratory for High-Efficiency Utilization of Solar Energy and Operation Control of Energy Storage System, Hubei University of Technology, Wuhan 430068, China; yfchen@hbut.edu.cn (Y.C.); 102410231@hbut.edu.cn (Q.G.); 102210252@hbut.edu.cn (J.Y.); 2 The School of Computer Science, Hubei University of Technology, Wuhan 430068, China

**Keywords:** myocardial infarction, S-transform, ECG, data augmentation

## Abstract

Accurate diagnosis of myocardial infarction (MI) holds significant clinical importance for public health systems. Deep learning-based ECG, classification and localization methods can automatically extract features, thereby overcoming the dependence on manual feature extraction in traditional methods. However, these methods still face challenges such as insufficient utilization of dynamic information in cardiac cycles, inadequate ability to capture both global and local features, and data imbalance. To address these issues, this paper proposes a ResNet-Transformer cascaded network (RTCN) to process time frequency features of ECG signals generated by the S-transform. First, the S-transform is applied to adaptively extract global time frequency features from the time frequency domain of ECG signals. Its scalable Gaussian window and high phase resolution can effectively capture the dynamic changes in cardiac cycles that traditional methods often fail to extract. Then, we develop an architecture that combines the Transformer attention mechanism with ResNet to extract multi-scale local features and global temporal dependencies collaboratively. This compensates for the existing deep learning models’ insufficient ability to capture both global and local features simultaneously. To address the data imbalance problem, the Denoising Diffusion Probabilistic Model (DDPM) is applied to synthesize high-quality ECG samples for minority classes, increasing the inter-patient accuracy from 61.66% to 68.39%. Gradient-weighted Class Activation Mapping (Grad-CAM) visualization confirms that the model’s attention areas are highly consistent with pathological features, verifying its clinical interpretability.

## Simple Summary

Myocardial infarction is a major cause of deaths worldwide, and electrocardiograms (ECGs) are key to diagnosis. Current ECG analysis has limitations such as overlooking dynamic cardiac cycle changes, failing to capture both local and global heart activity features, and being trained on imbalanced datasets. To address these issues, we developed a novel model that combines two deep neural networks, ResNet and Transformer. In addition, we introduced a method to generate more high-quality, samples from underrepresented classes. Our model performs well even with data from different patients. It also shows which parts of ECGs it uses for decisions, matching clinical signs. This helps doctors diagnose faster and more reliably, supporting better public health care.

## 1. Introduction

The number of deaths globally due to cardiovascular diseases (CVDs) in 2024 was approximately 18.6 million; this has been projected to rise to around 23.6 million by 2030. About 85% of CVD-related deaths are due to MI. Electrocardiograms (ECGs) are most suitable for the diagnosis of MI [1]. ECG abnormalities are detected through the deflections of the P, Q, R, S, and T waves or deviations in the intervals between these segments [2]. ECG interpretation by humans is time-consuming and highly subjective, especially in large-scale screening scenarios where its limitations are significant. Although deep learning technologies have driven advances in automated ECG analysis, existing methods mostly rely on single-domain features, such as time-domain waveforms, which makes it difficult to capture the multidimensional pathological features of MI [3]. Moreover, the class imbalance in MI datasets and the insufficient interpretability of models further hinder clinical translation.

Machine learning (ML) and deep learning (DL) have become dominant methodologies in MI classification and localization. Early studies primarily focused on traditional ML algorithms with manual feature extraction. For example, Hussein et al. [4] proposed an improved decision tree (DT) algorithm that optimizes branch structures and node-splitting strategies. Similarly, Li et al. [5] utilized a support vector machine (SVM) with feature engineering and parameter tuning to improve classification metrics. Although these methods achieved high accuracy, their reliance on handcrafted features introduced risks of information loss and misdiagnosis [6]. Subsequent studies have sought to address these limitations by integrating hybrid frameworks. Rahim et al. [7] developed MaLCaDD, that combines logistic regression with K-nearest neighbors (KNNs) to enhance generalization and stability. Rubini et al. [8] further validated the superiority of random forests (RFs) in handling complex medical data.

The advent of DL revolutionized MI classification and localization by enabling end-to-end learning from raw ECG signals [9,10]. Early DL models, such as the convolutional neural network–long short-term memory (CNN-LSTM) architecture by Rai et al. [11], demonstrated superior spatiotemporal feature extraction. Kwon et al. [12] and Liu et al. [13] expanded this by integrating multidimensional clinical parameters and multimodal data, achieving improved diagnostic specificity. Innovations like adaptive temporal networks [14] and dynamic input dimension adjustments [15] further optimized model robustness. For instance, Reasat et al. [16] achieved 96.96% accuracy using a CNN-based framework for inferior-wall MI detection, while Wang et al. [17] fused multilead ECG signals with triple subnetworks to enhance system reliability. Despite these advancements, early DL models lacked interpretability, limiting clinical adoption.

Recent work has addressed this gap. Jahmunah et al. [18] integrated Gradient-Weighted Class Activation Mapping (Grad-CAM) [19] to visualize decision-making regions in ECG signals. Han et al. [20,21] combined DL with knowledge graphs to provide explainable MI localization and severity predictions. Hybrid approaches, such as MaLCaDD [7] and interpretable DL frameworks, aim to address generalization issues. Concurrently, CNN-Transformer hybrids have been explored for biomedical signals. Liu et al. [22] embedded pyramid convolutions into U-shaped networks to replace self-attention, trading parameters for performance. Vindas et al. [23] employed a parallel CNN-Transformer with learnable late-fusion weights, achieving 99.7% PTB heartbeat accuracy yet requiring separate branch training and an extra fusion module. However, these methods still face many challenges, such as insufficient utilization of dynamic information in cardiac cycles, an inadequate ability to capture both global and local features, and data imbalance.

To address these issues, this paper proposes a ResNet-Transformer-Cascaded network (RTCN), incorporating multi-domain feature extraction, hybrid architecture design, and data augmentation strategies to improve the performance and clinical applicability of MI detection models. Different from the existing CNN-Transformer hybrids in biomedical signals, the proposed RTCN retains the full 50-layer ResNet as the local encoder, and cascades 3 Transformer layers directly. Instead of complex fusion in existing models, RTCN uses 1 × 1 conv to compress ResNet’s 7 × 7 × 512 tensor into 49 vectors, and injects learnable 2D positional embeddings. While existing models overfit on limited data, RTCN is end-to-end-trained from scratch. The main contributions of this work are as follows.

(1) S-transform is employed to convert ECG signals into high-resolution time frequency spectrograms. This enhances the representation of cardiac-cycle features and transient abnormalities, while retaining dynamic spectral features that boost MI-identification capability.

(2) A novel cascaded RTCN is proposed, which integrates the ability to extract the local morphological features of ResNet with the global temporal dependency modeling of Transformer. This design balances spatial granularity and temporal coherence, thereby improving model accuracy and interpretability.

(3) A data augmentation method based on DDPM is introduced to synthesize high-quality minority-class samples. This method effectively mitigates the data imbalance problem and enhances model robustness.

(4) Experiments on the PTB dataset show that the RTCN achieves accuracy rates of 99.79% and 68.39% under the intra-patient and inter-patient paradigms, respectively. After using DDPM for data augmentation, the overall model accuracy is enhanced from 61.66% to 68.39%. The F1 score is improved from 53.33% to 70.25%. Grad-CAM visualization verifies that the model’s attention areas are highly consistent with the pathological features extracted by S-transform. Experiments on the well-known PTB-XL dataset demonstrate that our method achieves significant performance improvements.

The structure of this paper is as follows. Section 2 describes the data preprocessing, S transform, and ResNet-Transformer-Cascaded framework. Section 3 presents the experimental results. Section 4 discusses the experimental results. Finally, Section 5 provides the conclusions.

## 2. Methods

### 2.1. Model Architecture Overview

**Figure 1:**
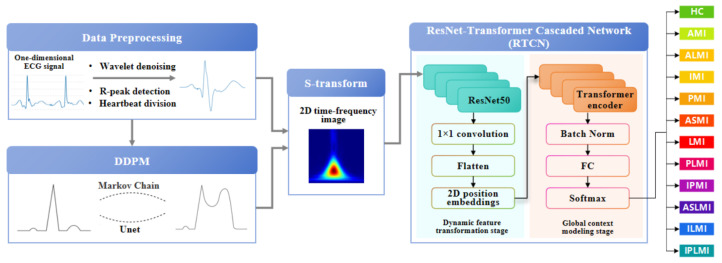
Overall diagram of the proposed model.

The RTCN aims to significantly enhance the robustness of MI detection by integrating local morphological features and global contextual associations across hierarchical levels [24]. As shown in Figure 1, the preprocessed one-dimensional ECG signal and the ECG samples after preprocessing in DDPM are transformed into two-dimensional time–frequency-domain images via S-transform [25]. In the multi-scale local perception stage, the generated two-dimensional time–frequency-domain image is fed into the ResNet50 network. The first four stages of ResNet50 are employed to extract multi-level feature maps, capturing detailed information from local to macroscopic scales. Following this, the feature maps undergo a series of transformations to prepare them for the subsequent stages. Specifically, the feature maps are first normalized using Batch Normalization to stabilize and accelerate the training process. They are then flattened into a one-dimensional vector to facilitate the application of fully connected layers. In the dynamic feature transformation stage, 1 × 1 convolution is utilized for channel dimension compression, and spatial features are flattened into sequential inputs. Simultaneously, learnable two-dimensional position embeddings are incorporated to preserve the original spatial topological priors. To prevent overfitting and enhance the model’s generalization capability, dropout is applied to randomly deactivate a fraction of neurons during training. In the global context modeling stage, the Transformer encoder processes the serialized features. The attention weights can explicitly quantify the contributions of different myocardial regions to lesion diagnosis, providing interpretable evidence for clinical practice. Finally, by introducing a learnable class token as a global semantic proxy, all positional features are aggregated and passed through a fully connected layer to output classification and localization probabilities. The Softmax function is applied to these probabilities to ensure they sum up to one, facilitating the interpretation as probabilities. This architecture enhances the discriminative expression of local features and strengthens the dynamic interaction of global information, achieving precise MI classification and localization.

To preclude patient-level data leakage, we applied a strict inter-patient split. All recordings were first deduplicated by unique patient ID, and then the entire set of patients was randomly divided into training and testing subsets at approximately an 8:2 ratio. Consequently, every ECG heartbeat from the same patient appears exclusively in either the training or the testing set, eliminating any patient-wise overlap that could inflate performance metrics.

### 2.2. Data Preprocessing

The original ECG signal contains a large amount of noise, such as electromyographic interference, baseline drift, and power-line interference [26]. It is necessary to remove the noise of the input ECG signal to avoid the influence of this noise on the classification and localization results. First, median filtering is employed to eliminate the baseline drift in the signal [27]. To separate the ECG signal from the noise, a threshold-based wavelet denoising method is adopted, as shown in Equation (1):

(1) $${\hat{d}}_{b}\left(c\right)=\left\{\begin{array}{cc} \mathrm{sgn}\left(d_{b}\left(c\right)\right)\left(\right|d_{b}\left(c\right)\left|-TE_{b}\right), & |d_{b}\left(c\right)|>TE_{b}\;\\ \;0, & |d_{b}\left(c\right)|\leq TE_{b} \end{array}\right.$$

where *b* = 1, …, 9, represents the number of levels of wavelet decomposition, and c denotes the number of sampling points in the signal. $TE_{b}$ is the set threshold, and its calculation method is as follows:

(2) $$TE_{b}=\left(\sigma_{b}\sqrt{2\log|d_{b}|}\right)/\log\left(b+1\right)$$

where $∥d_{b}∥$ is the two-norm of the wavelet coefficients, and $\sigma_{b}$ represents the estimated noise level. The calculation method is $\sigma_{b}=(median(∥d_{b}∥))/0.6745$ Here, 0.6745 is the value corresponding to the 75% area (with p = 0.25) of a Gaussian distribution with a mean of 0 and a variance of 1.

**Figure 2:**
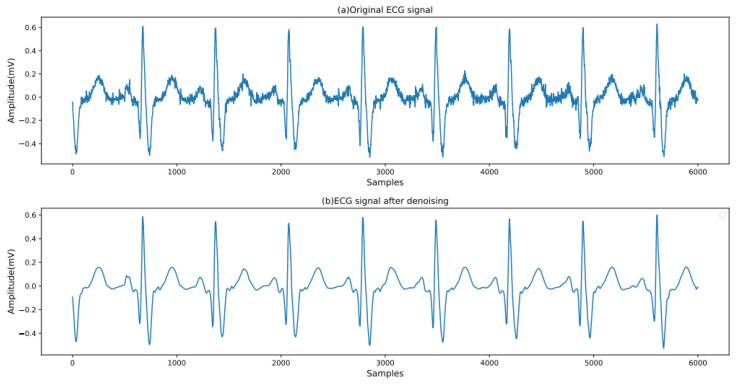
ECG signals before and after preprocessing.

Since the db6 wavelet has the same shape as the ECG signal, the db6 wavelet is selected as the wavelet basis. The signal is subjected to five-level wavelet decomposition. The wavelet coefficients that are smaller than $TE_{b}$ after decomposition are set to zero, thereby eliminating other noise signals. Subsequently, the Pan–Tompkins algorithm is employed to locate the QRS complex and the R peak [28]. A heartbeat is defined as the 250 sample points preceding the R peak and the 400 sample points following the R peak, with each ECG heartbeat containing 651 sample points in total. A comparison of the ECG signals before and after processing is shown in Figure 2.

### 2.3. Denoising Diffusion Probabilistic Model

**Figure 3:**
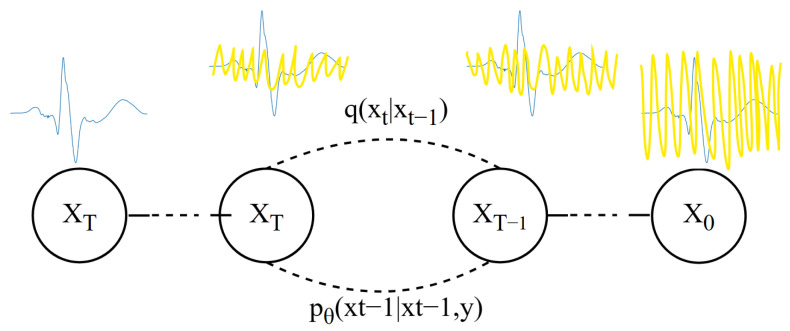
Schematic diagram of the DDPM diffusion process.

The DDPM [29] models the distribution of ECG data through a gradual process of adding and removing noise [30]. In the forward diffusion stage, the model starts from a clean ECG data sample and progressively adds noise until the ECG data becomes almost unrecognizable, reaching a highly stochastic state. This process can be viewed as the ECG data gradually “diffusing” into a high-entropy prior distribution [31]. The process is shown in Figure 3.

The forward diffusion formula is shown in Equation (3):

(3) $$q\left(x_{t}|x_{t-1}\right)=N\left(x_{t};\sqrt{1-\beta }x_{t-1},\beta_{t}I\right)$$

In the forward diffusion process, ${\left\{\beta_{t}\right\}}_{t=1}^{T}$ represents the variance used at each step, which ranges between 0 and 1. For diffusion models, the variance increases with the number of diffusion steps, satisfying $\beta_{1}<\beta_{2}<\ldots <\beta_{T}$. If the number of diffusion steps *T* is sufficiently large, the final $x_{T}$ will completely lose the original ECG data and become random noise. Each step of the diffusion process generates a noisy $x_{t}$, and the entire diffusion process forms a Markov chain, as shown in Equation (4):

(4) $$q\left(x_{1:t}|x_{0}\right)=\prod_{t=1}^{T}q\left(x_{t}|x_{t-1}\right)$$

In the reverse generation stage, the model learns how to gradually recover the original ECG data sample from this highly stochastic state, as shown in Equation (5):

(5) $$p_{\theta }\left(x_{0:t}\right)=p\left(x_{T}\right)\prod_{t=1}^{T}p\left(x_{t-1}|x_{t}\right)\;$$

Each denoising process is modeled as a Gaussian distribution:

(6) $$p_{\theta }\left(x_{t-1}|x_{t}\right)=N\left(x_{t-1}\right);\mu_{\theta }\left(x_{t},t\right),\sigma_{t}^{2}I\;$$

In Equation (6), $\mu_{\theta }$ is the mean predicted by the network, and $\mu_{\theta }\left(x_{t},t\right)$ is typically fixed at $\beta_{t}$ or ${\tilde{\beta }}_{t}=\frac{1-\alpha_{t-1}}{1-\alpha_{t}}$. The network is trained to progressively reduce the noise until the original ECG data sample is ultimately recovered. This process is opposite to forward diffusion and is thus referred to as “reverse generation”.

**Figure 4:**
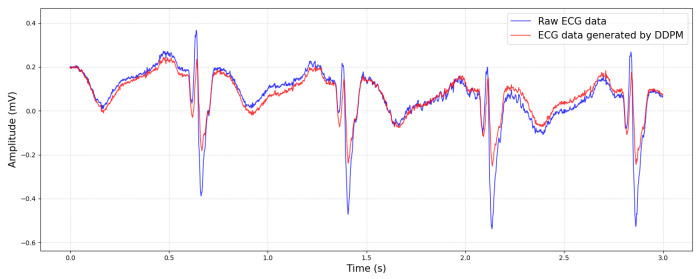
Comparison of ECG data generated by DDPM and original data.

Through training, the DDPM can learn the latent representation of the data distribution and generate high-quality new samples. This enables the DDPM to have a wide range of application prospects in fields such as image generation, audio synthesis, and time series prediction. A comparison between the generated ECG data and the original data is shown in Figure 4.

After augmenting the minority sample classes in the original data using the DDPM method, a more diverse and abundant set of samples was obtained. These augmented samples not only retained the key features of the original samples but also introduced new variations, enabling the model to better learn the underlying distribution of the samples during training. Meanwhile, for the majority sample classes in the original data, downsampling was performed to reduce their numerical dominance and prevent the model from being overly biased towards these classes during training. We initially balanced classes to a one-to-one ratio via downsampling the majority classes and DDPM-based generation for the minority classes. It is worth noting that all DDPM-generated samples retain the original ECG signal length to preserve clinical authenticity. However, due to inherent variations in original data length across different classes and the clinical requirement to retain complete ECG records per patient, perfect class balance was not achievable. We therefore fine-tuned the ratio by gradually adjusting the number of synthetic samples while evaluating validation performance, ultimately achieving an approximate balance across all twelve classes that preserved representative patterns from the original data distribution.

**Figure 5:**
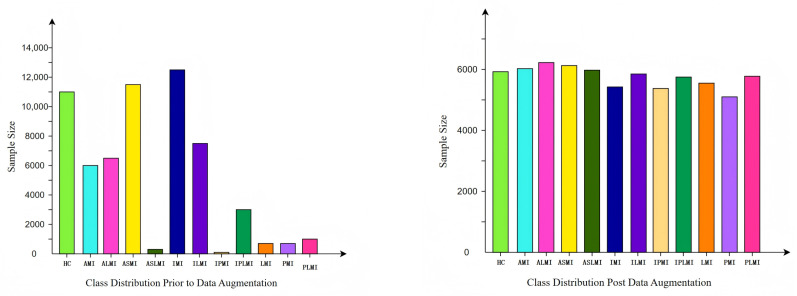
Data distribution before and after data augmentation.

The data distribution before and after augmentation is shown in Figure 5. It can be seen that the augmented data distribution is more balanced, with a significant reduction in the sample number differences between classes. This balanced data distribution helps the model to treat each class more fairly during training, thereby enhancing the model’s generalization ability.

The number of samples in the balanced PTB dataset [32] is shown in Table 1. As can be seen from the table, after data augmentation and downsampling, the number of samples in each category has become more consistent. The balanced data distribution not only effectively avoids problems such as overfitting but also enhances the model’s recognition performance on minority class samples.

### 2.4. Transformation Based on S-Transform

S-transform, as an extension of the continuous wavelet transform, offers several advantages, such as high-frequency resolution, the absence of cross-term interference, strong noise resistance, and adjustable window functions [33]. The S-transform of a signal x(t) is defined as follows:

(7) $$S\left(\tau ,f\right)=\int_{-\infty }^{+\infty }x\left(t\right)\frac{\left|f\right|}{\sqrt{2\pi }}e^{-\frac{{\left(\tau -t\right)}^{2}f^{2}}{2}}e^{-2i\pi ft}dt$$

Here, τ represents the time shift factor, and f denotes the frequency. The advantage of the S-transform in ECG analysis lies in its ability to provide more accurate time–frequency domain information. Compared with the short-time Fourier transform (STFT), the S-transform uses a scalable Gaussian window that can better adapt to frequency changes in the signal. Since the frequency of cardiac electrical activity varies over time, S-transform’s use of a Gaussian window with inverse frequency dependence allows it to more accurately capture this dynamic characteristic.

In this study, the parameters of S-transform are carefully chosen to optimize the time–frequency-domain representation of the ECG signals. The frequency resolution of S-transform refers to the interval between discrete points on the frequency axis. In our implementation, the frequency resolution is set to 1 Hz, meaning that the interval between each frequency point is 1 Hz. This setting ensures that S-transform can capture detailed frequency information across the entire frequency range of interest.

The window scaling in S-transform refers to the width of the Gaussian window function, which varies with frequency. Specifically, the width of the Gaussian window is inversely proportional to the frequency. As the frequency *f* increases, the window width decreases, making the Gaussian window narrower. Conversely, as the frequency *f* decreases, the window width increases, making the Gaussian window wider. This adaptive scaling allows the Gaussian window to be wider at low frequencies and narrower at high frequencies, providing good time resolution at high frequencies and good frequency resolution at low frequencies. This characteristic is crucial for capturing the dynamic changes in cardiac electrical activity, which are essential for accurate ECG analysis.

S-transform also offers better phase resolution, which is beneficial for detecting subtle changes in the signal. Given that ECG signals are typically complex and contain much noise, a tool with good phase resolution is required to extract useful information. Through phase correction and a scalable Gaussian window, S-transform can better extract features from the signal and provide a more accurate time–frequency-domain representation. Using S-transform, the dynamic characteristics and abnormal manifestations of cardiac electrical activity can be more accurately understood, thereby providing more reference information for clinical diagnosis and treatment [34].

**Figure 6:**
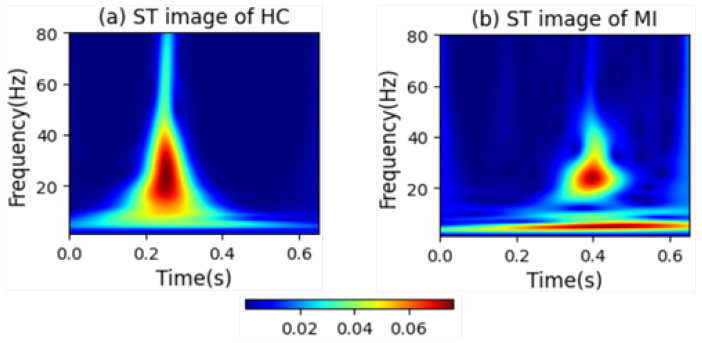
Images of heartbeats from MI and HC subjects after S-transform.

Figure 6 intuitively presents the differences in the frequency distribution of cardiac electrical activity between MI patients and HC subjects. For HC subjects, the images of their heartbeats after S-transform show a more regular and concentrated frequency distribution, with the main frequency components often concentrated within a relatively narrow time window. This reflects the stability and consistency of a normal cardiac rhythm.

In sharp contrast, the images of MI patients exhibit distinctly different characteristics. The main frequency appears around 0.4 s, and there is a significant shift in the main frequency distribution. The overall frequency distribution is more dispersed, with a large amount of low-frequency signals present throughout the entire cardiac cycle. The widespread presence of low-frequency signals may imply changes in the regulation function of the cardiac autonomic nervous system, which is manifested in the S-transform images as a broad and irregular frequency distribution.

### 2.5. ResNet-Transformer-Cascaded Network

**Figure 7:**
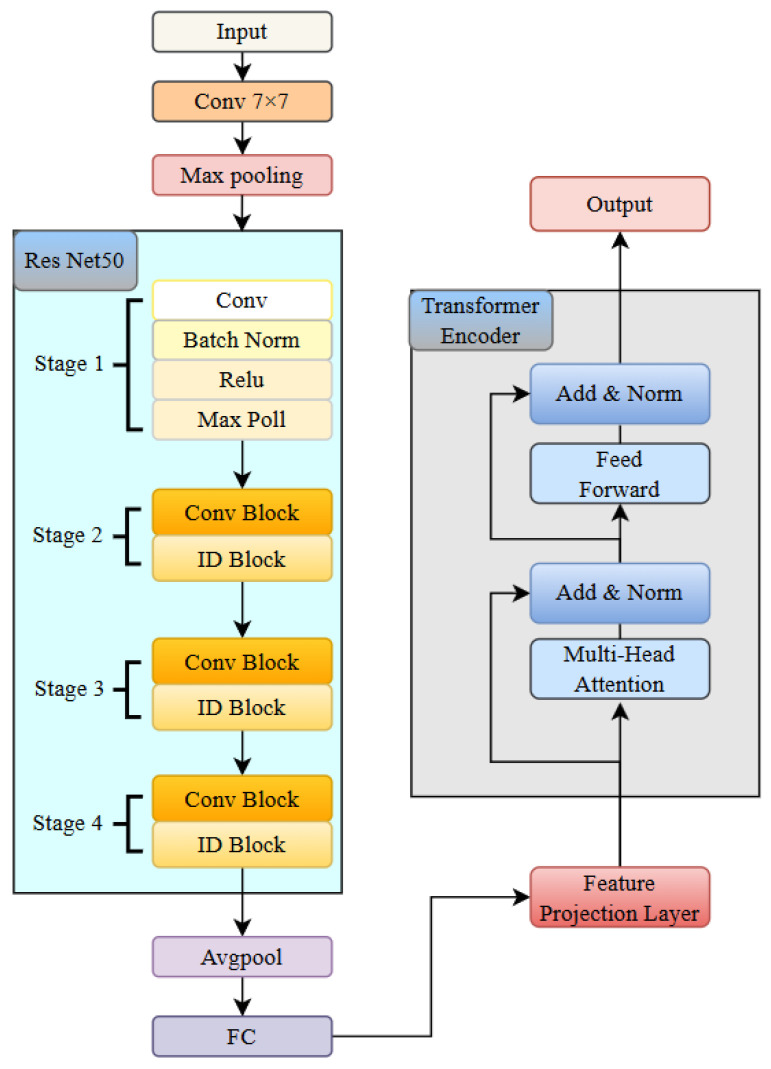
Structure diagram of ResNet-Transformer cascaded network.

The architecture of the RTCN is shown in Figure 7, which consists of a ResNet module and a Transformer encoder module [35]. The ResNet module consists of one 7 × 7 convolutional layer, one max pooling layer, and a backbone network composed of four groups of stacked residual blocks. Each group of residual blocks contains 3, 4, 6, and 3 residual units with a bottleneck structure, respectively, totalling 50 trainable weight layers. The network employs a cross-layer identity mapping design, with skip connections implementing the residual learning mechanism to effectively alleviate the gradient vanishing problem in deep networks [36]. The spatial dimensions of the feature maps in each stage are downsampled using a 1 × 1 convolution with a stride of 2. Batch Normalization (BN) and ReLU activation functions are used to enhance the stability of model convergence. The network parameter table is shown in Table 2, which details the kernel sizes, strides, output channels, and module repetition counts of each convolutional layer.

The Transformer encoder module includes the Self-Attention Mechanism and the Feed-Forward Neural Network [37]. In the Self-Attention Mechanism, each element in the input sequence can interact with other elements to calculate the correlation weights between them. These weights are used to dynamically adjust the contribution of each element to the final representation, enabling the model to focus on the most relevant information in the input sequence. The Feed-Forward Neural Network then performs further nonlinear transformations on each element to capture more complex features. In addition, Transformer encoders typically include a Positional Encoding mechanism, which provides positional information for each element in the sequence, as the Self-Attention Mechanism itself cannot process sequence order.

The proposed RTCN can achieve hierarchical fusion of local features and global context information through a cross-modal feature synergy mechanism. In the experiment, we first used the convolutional backbone network of ResNet50 to extract local spatial features from the S-transformed images, generating a high-level feature map with a resolution of 7 × 7 and a channel dimension of 2048. Then, we reduced the channel dimension from 2048 to 512 using a 1 × 1 convolution to reduce computational redundancy. Subsequently, we flattened the two-dimensional feature matrix along the spatial dimension into 49 embedding vectors, each with an embedding dimension of 512, and injected learnable two-dimensional relative positional encoding to preserve the global temporal information of MI. Finally, these serialized features were fed into the global relationship modeling module, composed of the Transformer encoder, which dynamically modeled the associations between different types of MI through multi-head attention weights. Meanwhile, a learnable class token was introduced as a global semantic proxy, and after aggregating all positional features, the classification and localization probability was output through a fully connected layer. In the MI classification and localization task, the model needs to capture global context information and local feature details. The learnable class token can better meet this requirement through dynamic interaction and a global semantic proxy. Through the multi-head attention mechanism of Transformer, the class token can dynamically interact with features at different positions and capture global dependencies. In addition, the class token is learnable, and the model can automatically adjust its weights according to the training data to better adapt to the task requirements. This architecture enhanced the discriminative expression of local features and the dynamic interaction of global context, achieving precise classification and pathological interpretation of MI.

The proposed RTCN architecture is designed to effectively integrate local morphological features and global temporal dependencies, addressing the limitations of existing deep learning models in capturing both aspects simultaneously. The ResNet module extracts multi-scale local features through its convolutional layers and residual connections, which are crucial for identifying detailed morphological abnormalities in ECG signals. The Transformer encoder, on the other hand, models long-range dependencies and global context, enhancing the model’s ability to understand the temporal dynamics of cardiac cycles. This combination not only improves the accuracy of myocardial infarction classification and localization but also provides a more comprehensive understanding of the underlying pathological features. The RTCN’s ability to balance spatial granularity and temporal coherence makes it a powerful tool for ECG analysis, offering significant advantages over traditional single-domain methods and other hybrid architectures.

## 3. Experimental Results

In this section, we evaluate and compare the classification and localization performance of the RTCN for different types of MI: intra-patient and inter-patient. Meanwhile, we reveal the attention areas of the model in recognizing different types of MI through Grad-CAM interpretability analysis. These areas are highly consistent with the ECG feature regions after S-transform.

### 3.1. Experimental Settings

During the training process, all models were trained using the cross-entropy loss function. Stochastic gradient descent with a learning rate of 0.001 and momentum of 0.9 was employed for parameter updates. The model’s loss is minimized through iterative steps, and its parameters are updated using error back-propagation. The batch size and number of epochs were set to 64 and 60, respectively. The experiments were conducted using Python 3.7. The deep learning program ran on the PyTorch 1.10.1 framework, utilizing NVIDIA GeForce RTX 4060 laptop GPUs to accelerate the training process. The experiments were conducted on a PC equipped with a 5.40 GHz Intel Core i9-13900HX CPU, 16 GB RAM, and a Windows 11 operating system.

### 3.2. Evaluation Indicators

To objectively and quantitatively compare the classification performance, we employed accuracy (Acc), sensitivity (Sen), precision (Pre), specificity (Spe), and the F1-Score to evaluate the classification results. These are defined as follows:

(8) $$Acc=\frac{TP+TN}{TP+FP+TN+FN},$$

(9) $$Sen=Recall=\frac{TP}{TP+FN},$$

(10) $$Pre=\frac{TP}{TP+FP},$$

(11) $$Spe=\frac{TN}{TN+FP},$$

(12) $$F_{1}-Score=\frac{2\times Sen\times Pre}{\left(Sen+Pre\right)},$$

TP and TN represent the number of true-positive and true-negative heartbeats, respectively. FN and FP denote the number of false negatives and false positives, respectively. The overall classification accuracy is defined as $Acc_{T}$ and is calculated as follows:

(13) $$Acc_{T}=\frac{\sum_{y=1}^{12}TP_{y}}{\sum_{y=1}^{12}TP_{y}+FN_{y}}$$

where $TP_{y}$ is the number of correctly detected heartbeats of each class, and $FN_{y}$ is the number of heartbeats of each class that were not correctly diagnosed.

### 3.3. Performance Verification of the Proposed RTCN

In this study, two distinct approaches were employed to evaluate the performance of the proposed RTCN: the intra-patient and inter-patient paradigms.

The intra-patient paradigm involves training and testing the model using ECG signals from the same patient, recorded at different time points or under different conditions. The intra-patient method ensures high consistency in feature distribution and noise levels, facilitating easier model learning and generalization.

The inter-patient paradigm involves training and testing the model using ECG signals from different patients. This approach more closely mimics real-world clinical scenarios, where ECG signals from different patients can exhibit significant variations in feature distribution and noise levels. While this method increases the difficulty of model training, it provides a more rigorous assessment of the model’s generalization ability. In practical clinical settings, physicians often encounter ECG signals from different patients, each with unique characteristics. The inter-patient method effectively simulates this scenario, allowing for a thorough evaluation of the model’s applicability in real-world clinical environments.

#### 3.3.1. Intra-Patient Experimental Results

**Figure 8:**
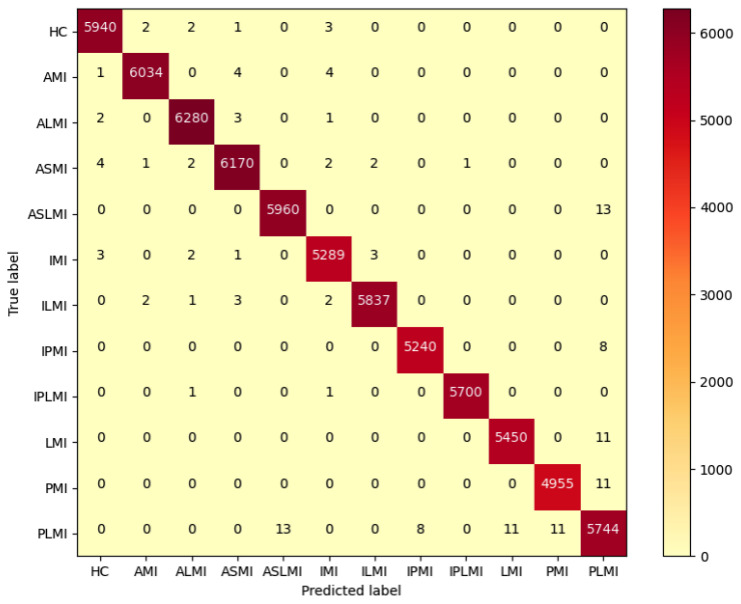
Confusion matrix of MI classification and localization by the RTCN in the intra-patient paradigm.

In the Intra-patient experiment, the confusion matrix for MI classification and localization by the model is shown in Figure 8, and the model’s ability to distinguish different MIs is shown in Table 3. Table 3 shows that the model performs outstandingly in predicting HC and AMI. The model achieved an accuracy of 99.97% and an F1 score of 99.85% for HC; for AMI, it achieved an accuracy of 99.98% and an F1 score of 99.88%, with a specificity of 99.99%. The overall classification and localization accuracy of the model for MI was 99.79%, demonstrating excellent performance. Moreover, the specificity of all MI types was stable above 99.87%, and the F1 scores were generally above 99.5%, fully validating the model’s high precision and strong robustness in the intra-patient paradigm for multi-class MI classification and localization tasks.

**Figure 9:**
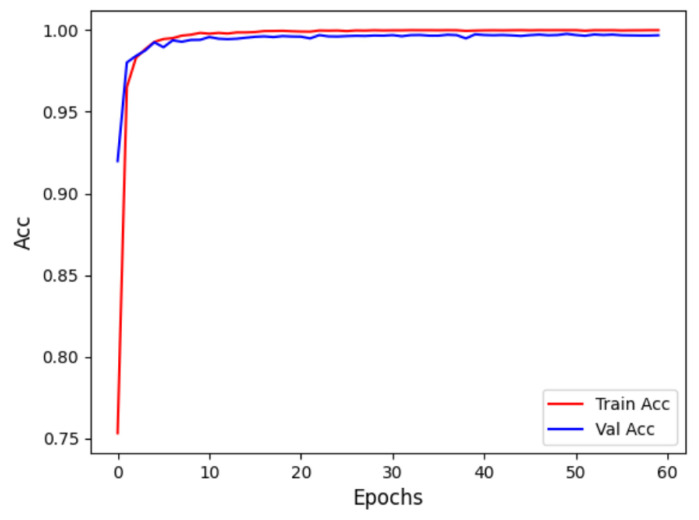
Intra-patient Acc curve.

**Figure 10:**
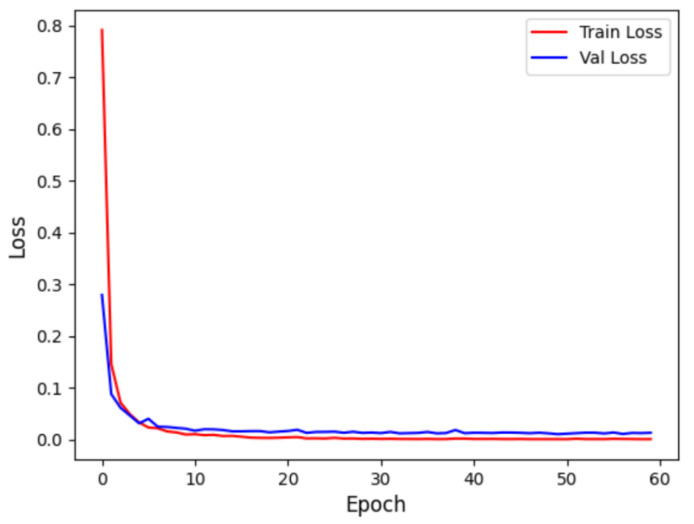
Intra-patient loss curve.

Figure 9 displays the accuracy curve during training and validation, while Figure 10 depicts the loss curve during training and validation, for the intra-patient experiment. It can be observed that the model converges quickly in the initial training phase, with training and validation accuracy close to 1, and the loss sharply decreases and then stabilizes. This indicates that the model did not experience overfitting and possesses strong generalization abilities.

#### 3.3.2. Inter-Patient Experimental Results

**Figure 11:**
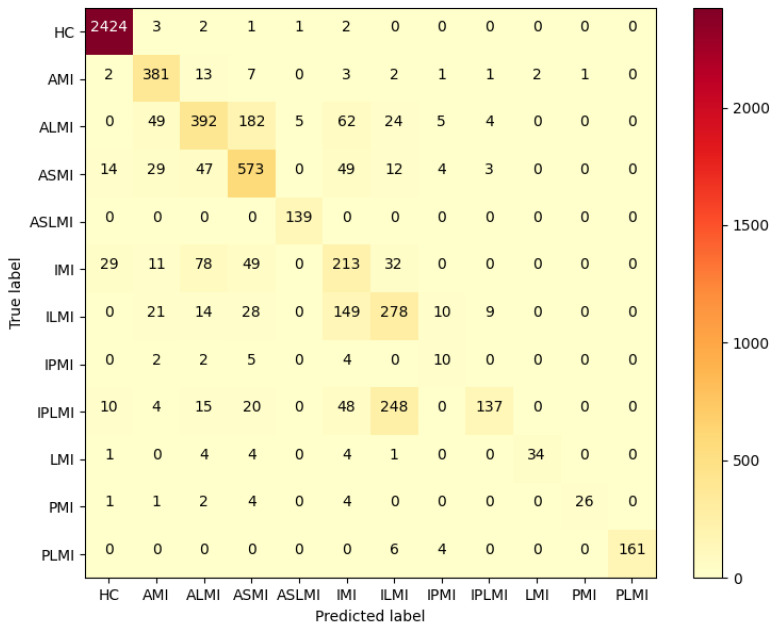
Confusion matrix of MI classification by the RTCN in the inter-patient paradigm.

For the inter-patient experiment, the confusion matrix for MI classification and localization by the model is shown in Figure 11, and the model’s classification ability for different MIs is shown in Table 4. Figure 11 shows that, although some heartbeats were misclassified in the Inter-patient paradigm, the majority of heartbeats in each class were correctly classified. As shown in Table 4, the F1 scores of the ALMI, IMI, ILMI, IPMI, and IPLMI were low. The average accuracy (ACC%) of the model was 96.37%.

### 3.4. Explainability Analysis

To understand the network’s ability to distinguish between different types of MI, we employ the Grad-CAM method. This technique visualizes the regions of interest in the input images that the network focuses on when making decisions. Grad-CAM is an interpretability technique for deep learning models that uses global average gradients to calculate the importance of each feature map for lass prediction and generates a heatmap by weighting and summing these feature maps.

**Figure 12:**
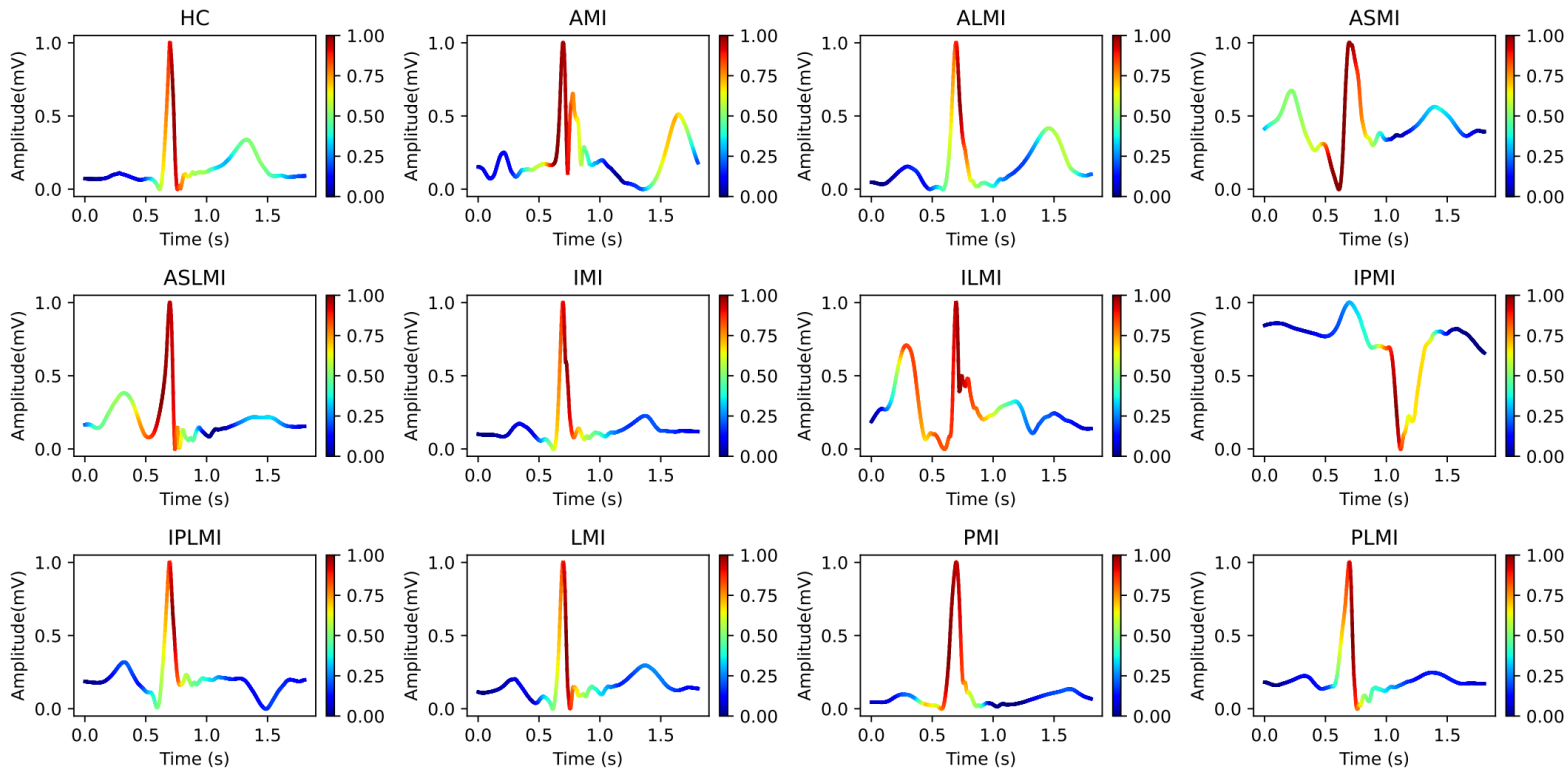
Heatmap of the attention distribution of the model in the time–frequency plane.

In this study, Grad-CAM heatmaps are generated from S-transformed two-dimensional time–frequency representations of ECG signals rather than from raw one-dimensional waveforms. This approach enables the model to identify critical features in both the time and frequency domains for accurate myocardial infarction localization. In Figure 12, the heatmaps illustrate the model’s attention across the time–frequency plane, with the x-axis representing time, the y-axis representing frequency components, and the color intensity indicating the importance of specific regions for decision-making.

To further enhance interpretability, we have incorporated Grad-CAM applied directly to the raw one-dimensional ECG signals. This allows for a comparative analysis between the attention mechanisms operating on the raw waveform and those derived from the time–frequency representations. We perform a detailed correlation analysis, demonstrating consistent attention around clinically significant segments, such as ST-segment deviations, in both representations. This strengthens the clinical relevance of our model’s decision-making process.

It is important to note that our study focuses on developing and interpreting an ECG-based deep learning model for MI localization. The statistical validation of attention regions is performed against ECG-derived features and clinically confirmed MI locations from the dataset annotations.

**Figure 13:**
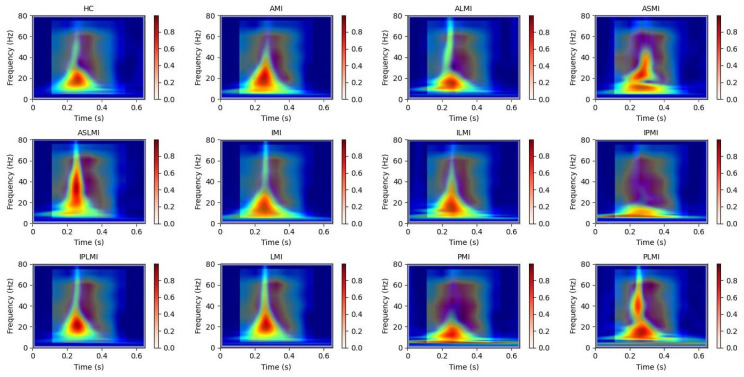
Interpretability images of the model’s intermediate layers using Grad-CAM.

**Figure 14:**
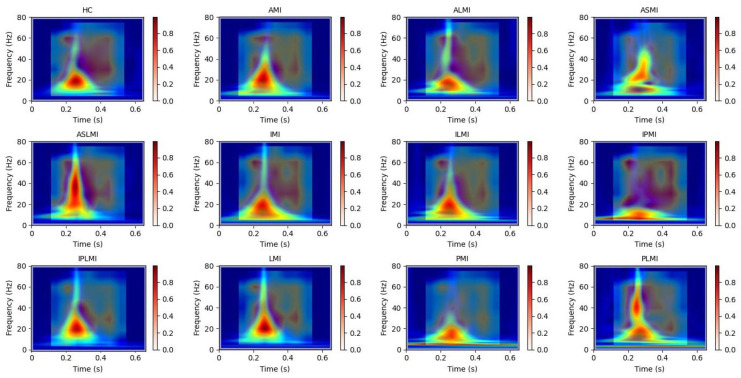
Interpretability images of the model’s output layer using Grad-CAM.

Our use of Grad-CAM to generate interpretability images of how the model distinguishes different MIs is shown in Figure 13 and Figure 14. It can be observed that the model focuses on different regions for different MI categories. Combining these with the images from S-transform, it can be seen that the model’s attention areas align highly with the ECG feature regions revealed by S-transform when identifying different MI types. For example, when identifying AMI, the model focuses on the significant changes in the main components of the QRS complex and the subsequent segment in the 5–15 Hz band of the S-transform image, which is consistent with the clinical ECG diagnostic criteria for AMI. This correspondence between the attention areas and the ECG features further validates the model’s effectiveness and reliability in MI identification and provides strong evidence for the model’s interpretability.

### 3.5. Ablation Studies

#### 3.5.1. Ablation Experiment on DDPM

To ensure the reliability of the experimental results, the inter-patient data division method is uniformly adopted in the ablation study.

To comprehensively evaluate the quality of ECG data generated by our Denoising Diffusion Probabilistic Model (DDPM), we employed three metrics: the Fréchet Inception Distance (FID), the Continuous Ranked Probability Score (CRPS), and Dynamic Time Warping (DTW). These metrics were selected to assess the distributional similarity, probabilistic calibration, amplitude accuracy, temporal fidelity, and morphological alignment of the generated ECG signals compared to real ECG signals.

(1) The FID was computed between the feature distributions of real ECG signals and synthetic signals generated by our DDPM model. Features were extracted using a pre-trained feature extractor, ensuring the evaluation captures high-level distributional similarities. A lower FID indicates superior distributional similarity.

(14) $$FID\left(r,g\right)=∥\mu_{r}-\mu_{g}{∥}_{2}^{2}+Tr\left(\Sigma_{r}+\Sigma_{g}-2{\left(\Sigma_{r}\Sigma_{g}\right)}^{1/2}\right)\;$$

(2) CRPS is calculated to assess the probabilistic calibration and amplitude-wise accuracy of the DDPM-generated signals compared to the real data. This metric is particularly valuable for evaluating the realism of the waveform’s amplitude variations. This integral calculates the square of the area between the generated empirical distribution CDF and the ideal CDF of the true values.

(15) $$CRPS\left(F,y^{t}\right)=\int_{-\infty }^{\infty }{\left(F\left(x\right)-1_{x\geq y^{t}}\right)}^{2}dx$$

The final CRPS score is the average of the CRPS values calculated at all time points *t* and for all test samples.

(16) $$\mathrm{Total}\;\mathrm{CRPS}=\frac{1}{M}\sum_{m=1}^{M}\frac{1}{T}\sum_{t=1}^{T}\mathrm{CRPS}\left(F_{m}^{t},y_{m}^{t}\right)$$

M is the number of test ECGs, and T is the length of the time points for each ECG. A lower CRPS value indicates that the empirical distribution generated by the DDPM is more concentrated and accurate, and its prediction uncertainty is better calibrated with the uncertainty of the real data. It measures the ability of the DDPM to capture the underlying distribution of the real ECG data.

(3) DTW is employed to quantify the temporal fidelity and morphological alignment between each DDPM-generated signal and its real counterpart. This directly measures how well our model preserves the critical temporal dynamics of ECG waveforms.

DTW is directly used to measure the morphological similarity between two specific ECG signals, ignoring their slight phase shifts on the time axis (such as a slight advance or delay in heartbeat). The DTW distance for each pair (real ECG, generated ECG) is calculated, and then the average or median of all paired distances is taken as an indicator of overall performance. A smaller DTW distance indicates that the generated ECG is more similar to the real ECG in morphology. It is very suitable for evaluating the similarity of time series (ECG), voice, and other time series that are insensitive to phase changes.

We conducted a comprehensive evaluation of the quality of ECG data generated by DDPM across different MI categories. As summarized in Table 5, the quantitative results demonstrate high fidelity in the synthesized signals: FID values range from 19.2 ± 2.1 to 24.5 ± 2.7, reflecting strong distributional alignment between real and generated samples across all infarction categories; CRPS values remain below 0.15 ± 0.017, indicating superior probabilistic calibration and minimal amplitude-wise deviations; and DTW measures are consistently under 10.8 ± 1.4, with relative deviations approximating 10–13% of the mean, confirming precise temporal dynamics alignment. These results collectively affirm the high quality and morphological integrity of the DDPM-generated ECG signals across all evaluated MI categories.

To evaluate the direct impact of DDPM data augmentation on model classification and localization performance, a single data augmentation experimental group was designed. In this experiment, the model was trained using data augmented by the DDPM, and compared with the original data without any data augmentation.

Table 6 shows the improvement effects of the DDPM data augmentation strategy on the RTCN model’s MI classification and localization performance under the inter-patient paradigm. After balancing the dataset with the DDPM, the overall model accuracy increased from 61.66% to 68.39%. The F1 score increased from 53.33% to 70.25%, and the overall accuracy of the RTCN model using the original dataset increased by 6.73%, verifying the promoting effect of data balancing on the model’s generalization ability. Figure 13 is the confusion matrix for MI classification and localization by the RTCN model using the original dataset under the inter-patient paradigm. It can be clearly seen that the RTCN model with the DDPM data augmentation strategy performs better in the classification and localization of minority MI classes.

#### 3.5.2. Ablation Experiment on Time-Frequency Methods

In the field of ECG signal analysis, various time–frequency analysis methods have been proposed to convert one-dimensional ECG signals into two-dimensional time–frequency representations. Among these methods, Continuous Wavelet Transform (CWT) and the Short-Time Fourier Transform (STFT) are widely used due to their ability to capture both time and frequency information. However, each method has its own strengths and limitations. For instance, STFT uses a fixed window size, which limits its ability to adapt to different frequency components, while CWT offers variable resolution but may not always provide the most intuitive connection to the Fourier spectrum.

Our choice of S-transform for this study was based on its unique properties that make it particularly advantageous for MI localization tasks:

(1) Superior Time–Frequency Resolution: Unlike STFT, which uses a fixed window size, S-transform employs a frequency-dependent window. This provides higher frequency resolution at lower frequencies, where most clinically relevant ECG components such as the P-wave, T-wave, and ST-segment reside, and higher time resolution at higher frequencies. This adaptive resolution is crucial for precisely pinpointing the subtle morphological changes caused by MI across different frequency bands. CWT also offers variable resolution, but S-transform can be considered a hybrid of STFT and WT, offering a more direct link to the Fourier spectrum.

(2) Phase Information Preservation: A key advantage of S-transform is that it retains the absolute phase information of the signal. This is not inherently provided by the magnitude scalograms generated by CWT, since the polarity and shape of ECG waves, such as ST-segment elevation or depression, are critical features for MI diagnosis and localization, preserving this phase information provides a richer feature set for the deep learning model to learn from.

To further validate our choice of the S-transform and to provide a comprehensive comparison, we conducted an additional ablation study. In this study, we compared the classification performance of our model using features extracted from STFT, CWT (using a Morlet wavelet), and S-transform representations. As shown in Table 7, our model achieves the highest F1-score and sensitivity when utilizing S-transform. This results support our initial design choice and highlights the effectiveness of S-transform in capturing the necessary features for MI localization.

#### 3.5.3. Ablation Experiment on Channel Dimension Compression

We conducted ablation studies, systematically varying the output dimension of the 1 × 1 convolutional layer while keeping all other model components and hyperparameters unchanged. The experiments were performed on the same hardware setup to ensure a fair comparison of computational latency, measured as the time taken to process a single ECG signal from input to final prediction. The comprehensive findings are presented in Table 8, revealing a clear trade-off between model performance and computational efficiency across varying dimensionalities. A reduction in dimensionality to 128 or 256 leads to a substantial decrease in computation time. However, this improvement in efficiency is accompanied by a pronounced reduction in classification accuracy and sensitivity. In contrast, increasing the dimensionality to 1024 or 1536 results in modest gains in performance metrics; for example, the F1-Score improves by 0.69% and 1.53%, respectively, compared to the 512-dimensional baseline. These enhancements, however, necessitate significantly greater computational resources, extending inference time by approximately two to threefold.

#### 3.5.4. Ablation Experiment on RTCN Model

To investigate the contribution of the Transformer structure to the RTCN model, we removed the Transformer encoder from the RTCN model, retaining only the ResNet50 backbone network, and conducted performance tests under the Inter-patient paradigm on the balanced dataset of PTB. As shown in Table 9, after removing the Transformer structure, the overall accuracy of the RTCN model decreased by 4.82% compared to the original 68.39%, and the F1 score also dropped from 70.25% to 60.34%. The confusion matrix in Figure 11 shows that, compared with the RTCN model, ResNet50 without the Transformer structure made more classification errors under the inter-patient paradigm on the dataset, indicating that the inclusion of the Transformer structure indeed enhanced the reliability of ResNet50’s comprehensive decision-making under the Inter-patient paradigm.

#### 3.5.5. Ablation Experiment on Transformer Model

To further evaluate the independent contribution of the Transformer structure, we have conducted an additional experiment using a standalone Vision Transformer (ViT) encoder. In this setup, the input S-transform time–frequency image is directly processed by the Transformer without the ResNet50 backbone. The image is split into fixed-size patches, which are then linearly embedded and fed into the same Transformer encoder architecture (3 layers, 8 heads), as used in our full model. This allows us to evaluate the Transformer’s capacity to model global spatial dependencies directly from the time–frequency representations.

We observe that the standalone Vision Transformer achieves moderate performance, outperforming ResNet50 alone in most metrics, particularly in Sensitivity (Sen) and F1-score. This indicates its strength in capturing global spatial features and long-range dependencies within the time–frequency images. However, it still underperforms compared to the full RTCN model, which synergistically combines the local feature extraction capability of ResNet50 with the global context modeling of the Transformer. This confirms that the two components are complementary and that their integration is beneficial.

As shown in Table 10, we observe that the standalone Vision Transformer achieves moderate performance, outperforming ResNet50 alone in most metrics, particularly in sensitivity (Sen) and F1-score. This indicates its strength in capturing global spatial features and long-range dependencies within the time–frequency images. However, it still underperforms compared to the full RTCN model, which synergistically combines the local feature extraction capability of ResNet50 with the global context modeling of the Transformer. This confirms that the two components are complementary and that their integration is beneficial.

### 3.6. Comparison of the Proposed Method with Other Methods

As shown in Table 11, the proposed method, the RTCN, in this paper is compared with existing MI localization methods. Only the ECG signals from lead 2 were used in this study. Compared with state-of-the-art methods, the RTCN increased MI localization accuracy under the inter-patient paradigm to 68.39%. Time-domain features are capable of capturing the morphological and temporal characteristics of ECG signals, providing the model with fundamental time-series information. Frequency-domain features focus on analyzing the spectral characteristics of the overall signal, revealing the distribution patterns of the signal in the frequency domain. Time–frequency domain features further reflect the dynamic changes in frequency over time and the instantaneous frequency characteristics, offering a more comprehensive and detailed signal description for the model. By integrating these multi-domain features, the model can extract richer and more diverse information from ECG signals, thereby enhancing the model’s accuracy and generalization ability.

## 4. Discussion

The experimental results demonstrate that the proposed RTCN significantly outperforms traditional single-domain analysis methods in the task of MI classification and localization by deeply integrating time–frequency features and multi-scale temporal dependencies. The RTCN overcomes the limitations of time-domain waveform feature representation and captures dynamic cardiac cycle features through the S-transform time–frequency spectrogram.

The proposed method achieved an accuracy rate of 99.79% and an average accuracy of 99.96% under the intra-patient paradigm of the PTB dataset. Under the inter-patient paradigm, the method achieved an accuracy of 68.39% when using a balanced dataset, which is 6.73% higher than that without balancing the dataset. This improvement highlights the significance of data balancing in enhancing model performance.

To gain a deeper understanding of the model’s decision-making process and validate its effectiveness, the Grad-CAM visualization technique was employed to analyze the time–frequency images generated by S-transform. The regions of interest identified by the model through Grad-CAM were found to be consistent with the key feature regions of S-transform, thereby verifying the model’s ability to accurately identify relevant features. This consistency underscores the model’s robustness and reliability in interpreting ECG signals.

In our study, we leveraged the integration of the DDPM, S-transform, and the RTCN to achieve enhanced performance in myocardial infarction classification and localization. The DDPM effectively addressed data imbalance by generating high-quality synthetic ECG samples, while the S-transform provided superior time–frequency resolution and phase information preservation. The RTCN architecture synergized local feature extraction from ResNet with global context modeling from the Transformer, resulting in precise and interpretable classifications.

In our hardware setup, the model was trained on a PC equipped with a 5.40GHz Intel Core i9-13900HX CPU, 16GB RAM, and an NVIDIA GeForce RTX 4060 GPU. The training process was efficient, with each epoch taking approximately 95 milliseconds, demonstrating the model’s computational feasibility.

However, our study has limitations. The dataset was limited in size and diversity, which might have affected the model’s generalizability. There are a few datasets that contain both MI classification and localization. The inter-patient experiments were challenging due to the significant variability in ECG signals across different patients. Additionally, the RTCN architecture can be further optimized for better performance and interpretability.

This study relies mainly on the publicly available PTB-XL database for model training and testing. Due to resource constraints, we are currently unable to acquire further large-scale, labeled, multi-center ECG data for external validation. The assessment of generalizability remains confined to PTB-XL and its subsets, which is a limitation of this work.

Another limitation is that our approach was only validated on standard 12-lead resting ECG, not multi-lead ambulatory or wearable ECG devices, critical for clinical continuous monitoring and daily health management. This is due to a lack of access to large, well-annotated ambulatory ECG datasets and ethical reviews needed to obtain wearable ECG data.

In the future, we plan to collaborate with cardiovascular hospitals to collect annotated ambulatory ECG data, and partner with wearable companies to build shared datasets, verifying our approach’s adaptability on these platforms to expand its application scope. Extending the approach to multi-modal signals, such as combining ECG with echocardiography or laboratory biomarkers, also holds significant potential. Echocardiography can provide structural or motion details of the heart, while lab biomarkers reflect myocardial injury at the molecular level. Integrating these via efficient fusion networks or cross-modal attention mechanisms is expected to notably improve the accuracy and robustness of early myocardial infarction detection and localization. We will also explore advanced network architectures and optimization techniques to improve model robustness and interpretability. Furthermore, we aim to develop cross-lead spatiotemporal attention modules to capture three-dimensional cardiac electrical activity features, enhancing the model’s clinical applicability.

In terms of network performance, future plans include exploring the use of larger ResNet models such as ResNet 101, ResNet 152, or ResNeXt models to improve performance, which is a promising direction.

## 5. Conclusions

This study proposes an MI diagnostic method based on S-transform and the RTCN. The S-transform time–frequency analysis effectively captures dynamic cardiac cycle features, enhancing the detection of transient ischemic patterns that are difficult for traditional time-domain methods to identify. The RTCN extracts local morphological abnormalities and global rhythm associations, increasing the model’s sensitivity to subtle pathological changes. The high-fidelity ECG samples generated by the DDPM alleviated the data imbalance issue. On the PTB dataset, the model achieved an accuracy of 99.79% and 68.39% in intra-patient and inter-patient validations, respectively. The DDPM data augmentation strategy significantly increased the inter-patient classification F1 score by 16.92%, and the recognition accuracy of minority classes by 6.73%. Grad-CAM visualization confirms that the model’s attention areas are highly consistent with clinical MI diagnostic markers, providing an interpretable physiological basis for algorithmic decision-making. Future work will focus on developing cross-lead spatiotemporal attention modules to capture three-dimensional cardiac electrical activity features and on optimizing lightweight diffusion models. In conclusion, our study facilitates bioinformatics research, addresses the issue of imbalanced biomedical data, improves MI diagnostic performance, and supports precision medicine and public health efforts.

## Figures and Tables

**Table 1:** Samples of the PTB dataset after data augmentation.

Types of MI	12-Lead Heartbeats
Anterior MI (AMI)	6043
Anterior Lateral MI (ALMI)	6286
Anterior Septal MI (ASMI)	6182
Anterior Septal Lateral MI (ASLMI)	5973
Inferior MI (IMI)	5298
Inferior Lateral MI (ILMI)	5845
Inferior Posterior MI (IPMI)	5248
Inferior Posterior Lateral MI(IPLMI)	5702
Lateral MI (LMI)	5461
Posterior MI (PMI)	4966
Posterior Lateral MI (PLMI)	5787
Healthy Control (HC)	5948
Total	68,739

**Table 2:** Network parameters of ResNet50.

Layer Name	ResNet50
Conv1	7 × 7 × 64, stride 2
Conv2_x	3 × 3, max pool, stride 2
	$\left[\begin{array}{c} 1\times 1,64\\ 3\times 3,64\\ 1\times 1,256 \end{array}\right]\times 3$
Conv3_x	$\left[\begin{array}{c} 1\times 1,128\\ 3\times 3,128\\ 1\times 1,512 \end{array}\right]\times 4$
Conv4_x	$\left[\begin{array}{c} 1\times 1,256\\ 3\times 3,256\\ 1\times 1,1024 \end{array}\right]\times 6$
Conv5_x	$\left[\begin{array}{c} 1\times 1,512\\ 3\times 3,512\\ 1\times 1,2048 \end{array}\right]\times 3$
	Average pool,
	fc, softmax

**Table 3:** Specific performance of the RTCN on various MIs in the intra-patient paradigm.

Class	${\mathrm{Acc}}_{T}$ (%)	Sen (%)	Pre (%)	Spe (%)	F1 (%)
HC	99.97	99.87	99.83	99.98	99.85
AMI	99.98	99.85	99.95	99.99	99.88
ALMI	99.98	99.90	99.98	99.99	99.89
ASMI	99.97	99.81	99.99	99.98	99.81
ASLMI	99.96	99.78	99.64	99.98	99.78
IMI	99.97	99.83	99.75	99.98	99.79
ILMI	99.98	99.86	99.91	99.99	99.89
IPMI	99.98	99.85	99.85	99.99	99.85
IPLMI	100	99.96	99.98	100	99.97
LMI	99.97	99.80	99.80	99.98	99.80
PMI	99.97	99.78	99.78	99.98	99.78
PLMI	99.87	99.26	99.26	99.93	99.26
Average	99.96	99.83	99.85	99.98	99.84

**Table 4:** Specific performance of the RTCN on various MIs in the inter-patient paradigm.

Class	${\mathrm{Acc}}_{T}$ (%)	Sen (%)	Pre (%)	Spe (%)	F1 (%)
HC	98.92	99.63	98.45	97.70	98.66
AMI	97.52	92.25	97.90	76.05	83.37
ALMI	91.70	54.22	96.72	68.89	60.68
ASMI	92.52	78.39	94.44	65.64	71.45
ASLMI	99.90	100	99.90	95.86	97.89
IMI	91.44	51.70	94.31	39.59	44.84
ILMI	90.92	54.62	94.21	46.10	50.00
IPMI	99.40	43.48	99.61	29.41	35.09
IPLMI	94.09	28.42	99.70	88.96	43.08
LMI	99.74	70.83	99.97	94.44	80.95
PMI	99.79	68.42	99.98	96.30	80.00
PLMI	99.84	94.15	100	100	96.99
Average	96.37	72.83	97.49	74.63	72.98

**Table 5:** Quantitative validation for quality of synthetic ECG generated by DDPM.

Class	FID	CRPS	DTW
HC	192.2 ± 2.1	0.115 ± 0.012	7.8 ± 0.9
AMI	21.5 ± 2.3	0.128 ± 0.014	9.1 ± 1.1
ALMI	22.1 ± 2.4	0.132 ± 0.015	9.5 ± 1.2
ASMI	20.8 ± 2.2	0.126 ± 0.013	8.9 ± 1.0
ASLMI	23.0 ± 2.5	0.135 ± 0.015	10.2 ± 1.3
IMI	21.0 ± 2.3	0.130 ± 0.014	9.3 ± 1.1
ILMI	22.5 ± 2.4	0.134 ± 0.015	9.8 ± 1.2
IPMI	24.1 ± 2.6	0.140 ± 0.016	10.5 ± 1.4
IPMI	23.8 ± 2.6	0.138 ± 0.016	10.3 ± 1.3
IPLMI	22.2 ± 2.4	0.133 ± 0.015	9.6 ± 1.2
PMI	24.5 ± 2.7	0.142 ± 0.017	10.8 ± 1.4
PLMI	23.5 ± 2.6	0.137 ± 0.016	10.1 ± 1.3

**Table 6:** Performance comparison of RTCN under the inter-patient paradigm before and after DDPM data augmentation.

Method	${\mathrm{Acc}}_{T}$ (%)	Sen (%)	Pre (%)	Spe (%)	F1 (%)
DDPM-balanced dataset	68.39	69.68	74.91	97.93	70.25
Raw datasets	61.66	36.74	43.08	96.38	53.33

**Table 7:** Performance comparison of different time–frequency representations.

Method	${\mathrm{Acc}}_{T}$ (%)	Sen (%)	Pre (%)	Spe (%)	F1 (%)
S-transform	68.39	69.68	74.91	97.93	70.25
CWT	66.74	67.21	72.35	97.65	68.12
STFT	64.18	63.89	70.14	97.42	65.72

**Table 8:** Performance and computation time under different dimensionality reduction levels in inter-patients.

Reduced Dimension	${\mathrm{Acc}}_{T}$ (%)	Sen (%)	Pre (%)	Spe (%)	F1 (%)	CPU Time (ms)
1536	62.35	38.12	44.28	96.52	54.18	∼285
1024	61.97	37.58	43.75	96.45	53.72	∼190
512	61.66	36.74	43.08	96.38	53.33	∼95
256	60.25	35.36	42.15	96.05	52.21	∼48
128	58.83	34.07	41.22	95.72	50.89	∼24

**Table 9:** Performance comparison of RTCN and ResNet50 under the inter-patient paradigm.

Data Set	${\mathrm{Acc}}_{T}$ (%)	Sen (%)	Pre (%)	Spe (%)	F1 (%)
RTCN	68.39	69.68	74.91	97.93	70.25
ResNet50	63.57	47.26	62.83	96.89	60.34

**Table 10:** Comparison of the performance of a single Transformer under the inter-patient paradigm.

Method	${\mathrm{Acc}}_{T}$ (%)	Sen (%)	Pre (%)	Spe (%)	F1 (%)
RTCN	68.39	69.68	74.91	97.93	70.25
ResNet50	63.57	47.26	62.83	96.89	60.34
Transformer	65.82	58.41	66.50	97.20	61.90

**Table 11:** Comparison of the proposed method with other methods.

Methods	Leads and Database	Location	Intra-Patient	Inter-Patient
CNN based on ResNet [38]	12 leads PTB	Location	Acc = 99.72% Se = 99.63%	Acc = 55.74% Se = 47.58%
CNN and BiGRU with attention [39]	12 leads PTB	Location	Acc = 99.11% Se = 99.02%	Acc = 62.94% Se = 63.97%
Multi-scale feature [40]	12 leads PTB	Location	—	Acc = 61.82%
DenseNet [41]	12 leads PTB	Location	Acc = 99.87% Se = 99.84% SP = 99.98%	—
Multi-scale ResNet with attention [42]	12 leads PTB	Location	Acc = 99.79% Se = 99.88%	—
Tucker2 decomposition [43]	12 leads PTB	Location	Acc = 99.67% Se = 99.98% SP = 99.82%	Acc = 65.11% Se = 98.29% SP = 71.91%
Multi-lead branch with ResNet with SE and LSTM [21]	12 leads PTB	Location	Acc = 99.69% Se = 99.58%	Acc = 67.89% Se = 63.16%
RTCN	Lead II	Location	Acc = 99.79% Se = 99.84% SP = 99.98%	Acc = 68.39% Se = 69.68% SP = 97.93%

## Data Availability

The datasets analyzed in this study are publicly available. ECG data were obtained from the PTB Diagnostic ECG Database and PTB-XL database, accessible via PhysioNet (https://physionet.org/content/ptbdb/1.0.0/, accessed on 25 September 2004) and Physikalisch-Technische Bundesanstalt (https://physionet.org/content/ptb-xl/1.0.3/, accessed on 9 November 2022), respectively. Derived data supporting the results can be provided upon reasonable request to the corresponding author.
